# Detection of COVID-19 using multimodal data from a wearable device: results from the first TemPredict Study

**DOI:** 10.1038/s41598-022-07314-0

**Published:** 2022-03-02

**Authors:** Ashley E. Mason, Frederick M. Hecht, Shakti K. Davis, Joseph L. Natale, Wendy Hartogensis, Natalie Damaso, Kajal T. Claypool, Stephan Dilchert, Subhasis Dasgupta, Shweta Purawat, Varun K. Viswanath, Amit Klein, Anoushka Chowdhary, Sarah M. Fisher, Claudine Anglo, Karena Y. Puldon, Danou Veasna, Jenifer G. Prather, Leena S. Pandya, Lindsey M. Fox, Michael Busch, Casey Giordano, Brittany K. Mercado, Jining Song, Rafael Jaimes, Brian S. Baum, Brian A. Telfer, Casandra W. Philipson, Paula P. Collins, Adam A. Rao, Edward J. Wang, Rachel H. Bandi, Bianca J. Choe, Elissa S. Epel, Stephen K. Epstein, Joanne B. Krasnoff, Marco B. Lee, Shi-Wen Lee, Gina M. Lopez, Arpan Mehta, Laura D. Melville, Tiffany S. Moon, Lilianne R. Mujica-Parodi, Kimberly M. Noel, Michael A. Orosco, Jesse M. Rideout, Janet D. Robishaw, Robert M. Rodriguez, Kaushal H. Shah, Jonathan H. Siegal, Amarnath Gupta, Ilkay Altintas, Benjamin L. Smarr

**Affiliations:** 1 Osher Center for Integrative Health, University of California San Francisco, San Francisco, CA USA; 2 MIT Lincoln Laboratory, Massachusetts Institute of Technology, Lexington, MA USA; 3 Halıcıoğlu Data Science Institute, University of California San Diego, La Jolla, CA USA; 4 Department of Biomedical Informatics, Harvard Medical School, Boston, MA USA; 5 Department of Management, Zicklin School of Business, Baruch College, The City University of New York, New York, NY USA; 6 San Diego Supercomputer Center, University of California San Diego, San Diego, CA USA; 7 Department of Electrical and Computer Engineering, University of California San Diego, La Jolla, CA USA; 8 Department of Bioengineering: Bioinformatics, University of California San Diego, San Diego, CA USA; 9 Department of Psychology, Drexel University, Pennsylvania, PA USA; 10 Vitalant Research Institute, University of California San Francisco, San Francisco, CA USA; 11 Department of Psychology, University of Minnesota - Twin Cities, Minneapolis, MN USA; 12 Love School of Business, Elon University, Elon, NC USA; 13 School of Medicine, University of California San Francisco, San Francisco, CA USA; 14 Department of Anesthesiology, Northwestern McGaw Medical Center, Feinberg School of Medicine, Chicago, IL USA; 15 Department of Emergency Medicine, University of California Los Angeles Health, Los Angeles, CA USA; 16 Center for Health and Community, University of California San Francisco, San Francisco, CA USA; 17 Department of Emergency Medicine, Beth Israel Deaconess Medical Center Boston, Boston, MA USA; 18 Schmidt College of Medicine, Florida Atlantic University, Boca Raton, FL USA; 19 Department of Neurosurgery, Santa Clara Valley Medical Center, Stanford University, San Jose, CA USA; 20 Department of Emergency Medicine, Jamaica Hospital Medical Center, Jamaica, NY USA; 21 Department of Emergency Medicine, Boston Medical Center, Boston, MA USA; 22 Department of Anesthesiology: Pain Management and Perioperative Medicine, University of Miami, Miami, FL USA; 23 Department of Emergency Medicine, New York Presbyterian Brooklyn Methodist Hospital, Brooklyn, NY USA; 24 Department of Anesthesiology and Pain Management, University of Texas Southwestern, Dallas, TX USA; 25 Department of Biomedical Engineering, Renaissance School of Medicine, Stony Brook University, Stony Brook, NY USA; 26 Stony Brook Medicine, Stony Brook University Renaissance School of Medicine, Stony Brook, NY USA; 27 Department of Anesthesia: Perioperative and Pain Medicine, Kaiser Permanente San Diego, San Diego, CA USA; 28 Department of Emergency Medicine, Tufts Medical Center, Boston, MA USA; 29 Department of Emergency Medicine, University of California San Francisco, San Francisco, CA USA; 30 Weill Cornell Medical Center, Weill Cornell Medical School, New York, NY USA; 31 New York Presbyterian Queens, Weill-Cornell Medical College, Queens, NY USA

**Keywords:** Biotechnology, Medical research, Signs and symptoms, Diagnostic markers, Predictive markers, Scientific data

## Abstract

Early detection of diseases such as COVID-19 could be a critical tool in reducing disease transmission by helping individuals recognize when they should self-isolate, seek testing, and obtain early medical intervention. Consumer wearable devices that continuously measure physiological metrics hold promise as tools for early illness detection. We gathered daily questionnaire data and physiological data using a consumer wearable (Oura Ring) from 63,153 participants, of whom 704 self-reported possible COVID-19 disease. We selected 73 of these 704 participants with reliable confirmation of COVID-19 by PCR testing and high-quality physiological data for algorithm training to identify onset of COVID-19 using machine learning classification. The algorithm identified COVID-19 an average of 2.75 days before participants sought diagnostic testing with a sensitivity of 82% and specificity of 63%. The receiving operating characteristic (ROC) area under the curve (AUC) was 0.819 (95% CI [0.809, 0.830]). Including continuous temperature yielded an AUC 4.9% higher than without this feature. For further validation, we obtained SARS CoV-2 antibody in a subset of participants and identified 10 additional participants who self-reported COVID-19 disease with antibody confirmation. The algorithm had an overall ROC AUC of 0.819 (95% CI [0.809, 0.830]), with a sensitivity of 90% and specificity of 80% in these additional participants. Finally, we observed substantial variation in accuracy based on age and biological sex. Findings highlight the importance of including temperature assessment, using continuous physiological features for alignment, and including diverse populations in algorithm development to optimize accuracy in COVID-19 detection from wearables.

## Introduction

The COVID-19 pandemic highlighted gaps in the public health responses to transmissible disease epidemics. If continuous, passive screening tools for infectious diseases such as COVID-19 could be developed and deployed, they may hold potential to substantially reduce the spread and impact of disease by assisting individuals in recognizing when they should self-isolate, seek testing, and possibly obtain early medical intervention. Consumer wearable devices that continuously measure physiological metrics such as dermal temperature, heart rate, and respiratory rate can establish users’ individual baseline patterns and allow detection of deviations from their baselines. Because these physiological variables can change in response to infection^1–3^ consumer wearables may hold promise as broadly available tools for early illness detection. Previous studies have shown that wearable devices can collect physiological signals that predict or correlate with SARS-CoV-2 infection^4–7^. Earlier studies have also demonstrated that such technology holds promise in predicting influenza-like illnesses^8^. Though promising, these studies highlight challenges in how to train illness-detection algorithms with limited labels such as date of diagnosis, which may not correspond well to onset of physiological disruption. Additionally, it is not yet clear which sensors are most useful in illness onset. Previous studies did not collect data from wearables that measure dermal temperature, because such sensors were not widely available on wearable devices. Including this latter measure may improve infection detection, as a key response to infectious diseases is change in body temperature.

## Results

### Study overview

**Figure 1:**
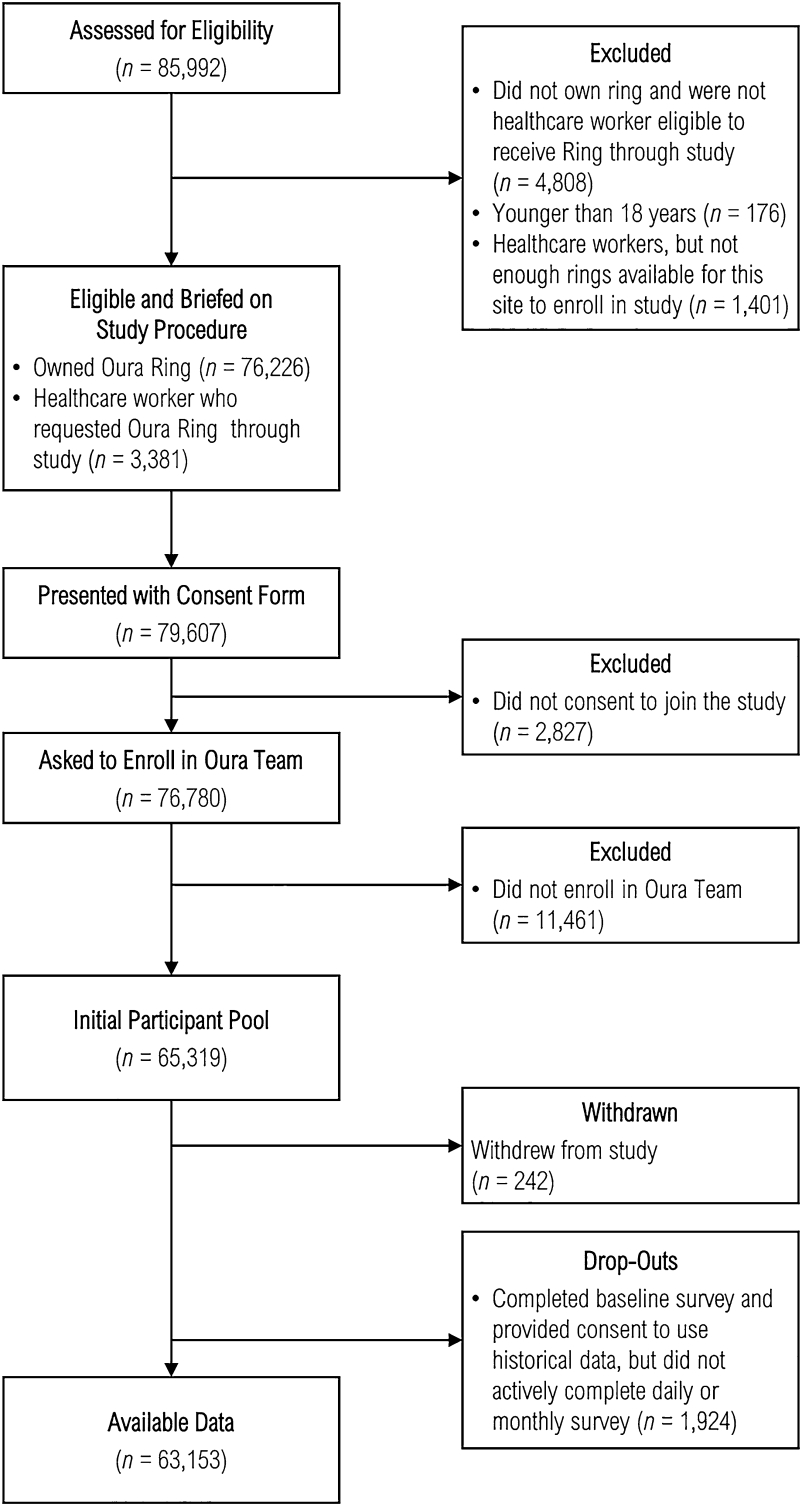
Enrollment and follow-up.

We initiated the TemPredict Study in March of 2020 to assess whether off-the-shelf wearable devices collect data that could be used to screen large numbers of individuals for the early stages of SARS-CoV-2 infection. All participants wore a commercially available smart-ring wearable device (Oura Ring) that pairs with a smartphone app, completed daily surveys of symptoms and COVID-19 diagnoses, and completed a baseline survey and longer monthly surveys. The Oura Ring collects dermal temperature, uses photoplethysmography (PPG) data to measure heart rate, heart rate variability, and respiratory rate, and estimates physical activity based on accelerometry data (recorded as metabolic equivalents; METs). TemPredict enrolled individuals who already owned an Oura Ring and who responded to an in-app invitation to participate (*n* = 73,399), of whom 62,139 met all inclusion criteria. TemPredict also enrolled healthcare workers (*n* = 3,180) at participating settings and provided healthcare workers with Oura Rings for study participation. Of 65,319 individuals in the initial participant pool, 242 withdrew participation, and 1,924 did not engage sufficiently with study activities to be included in analyses (i.e., completed the baseline survey, but did not complete any of the daily or monthly follow up surveys), resulting in 63,153 participants (see Fig. 1 and Table 1).

**Table 1:** Participant characteristics.

Characteristic	Overall*, *N* = 63,153	COVID-19 Training*, *n* = 73	COVID-19 Independent Validation*, *n* = 10
**Age, n (%)**			
18–30 years	8,555 (14%)	9 (12%)	1 (10%)
31–40 years	16,756 (27%)	22 (31%)	1 (10%)
41–50 years	17,502 (29%)	18 (25%)	3 (30%)
51–80 years	18,148 (30%)	23 (32%)	5 (50%)
81 + years	102 (0.2%)	0 (0%)	0 (0%)
**Sex, n (%)**			
Female	24,374 (40%)	29 (40%)	2 (20%)
Male	36,632 (60%)	43 (60%)	8 (80%)
Other	56 (< 0.1%)	0 (0%)	0 (0%)
**Race, n (%)**			
Non-Hispanic White	50,130 (82%)	61 (85%)	9 (90%)
Non-Hispanic Black	909 (1.5%)	1 (1.4%)	0 (0%)
African	107 (0.2%)	0 (0%)	0 (0%)
American Indian	98 (0.2%)	0 (0%)	0 (0%)
Native Hawaiian or Other Pacific Islander	161 (0.3%)	0 (0%)	0 (0%)
Asian	3,313 (5.4%)	1 (1.4%)	0 (0%)
South Asian	936 (1.5%)	0 (0%)	0 (0%)
Middle Eastern	595 (1.0%)	1 (1.4%)	1 (10%)
Other	4,768 (7.8%)	8 (11%)	0 (0%)
**Ethnicity, n (%)**			
Hispanic or Latino Origin	3,570 (5.8%)	11 (15%)	0 (0%)
Non-Hispanic	57,491 (94%)	61 (85%)	10 (100%)
**Education, n (%)**			
Less than high school	284 (0.5%)	0 (0%)	0 (0%)
High School/GED or some college	7,958 (13%)	13 (18%)	1 (10%)
Associate degree or higher	51,754 (85%)	58 (81%)	9 (90%)
Didn't specify	1,066 (1.7%)	1 (1.4%)	0 (0%)
**Frontline Workers, n (%)**	7,810 (13%)	8 (11%)	2 (20%)
**# Comorbid Conditions, n (%)**			
0	53,222 (87%)	69 (96%)	10 (100%)
1	5,269 (8.6%)	2 (2.8%)	0 (0%)
2 or more	2,524 (4.1%)	1 (1.4%)	0 (0%)
**Mean # of Daily Symptoms**^**1**^**, n (%)**			
No Symptoms	(n/a)	8 (12%)	0 (0%)
1 to 3 Symptoms	(n/a)	33 (48%)	1 (12%)
4 to 6 Symptoms	(n/a)	24 (35%)	7 (88%)
Greater than 6 Symptoms	(n/a)	4 (5.8%)	0 (0%)
**Antibody Tests, n (%)**			
1 or more tests	8,736 (14%)	33 (45%)	10 (100%)
No tests	54,417 (86%)	40 (55%)	0 (0%)
**Antibody Test Results, n (%)**			
Non-reactive	7,710 (88%)	6 (18%)	0 (0%)
Reactive	175 (2.0%)	18 (55%)	10 (100%)
Indeterminate	851 (9.7%)	9 (27%)	0 (0%)

*n (% of non-missing). ^1^mean number of daily symptoms were computed for period spanning 3 days before to 3 days after the diagnosis date (for participants with a confirmed diagnosis). In the overall sample, *n* = 61,063 had available age data; *n* = 61,062 had available sex data, education data, and frontline worker status data; *n* = 61,017 had available race data; *n* = 61,061 had available ethnicity data; and *n* = 61,015 had available comorbid condition data. Within COVID-19 training data, *n* = 72 had available data for age, sex, race, ethnicity, education, frontline worker status, and comorbid conditions; *n* = 69 had available data for symptoms. Within COVID-19 independent validation data *n* = 8 had available data for symptoms; *n* = 10 had available data for all other characteristics.

Of the 63,153 participants with available Oura Ring data, we identified 704 who reported on a study survey that they may have had COVID-19, 306 of whom reported that they had been diagnosed with COVID-19 using a reliable laboratory test. Of these 306, we selected 73 with the most complete Oura Ring and daily symptom survey data to use for algorithm training.

### Algorithm development

**Figure 2:**
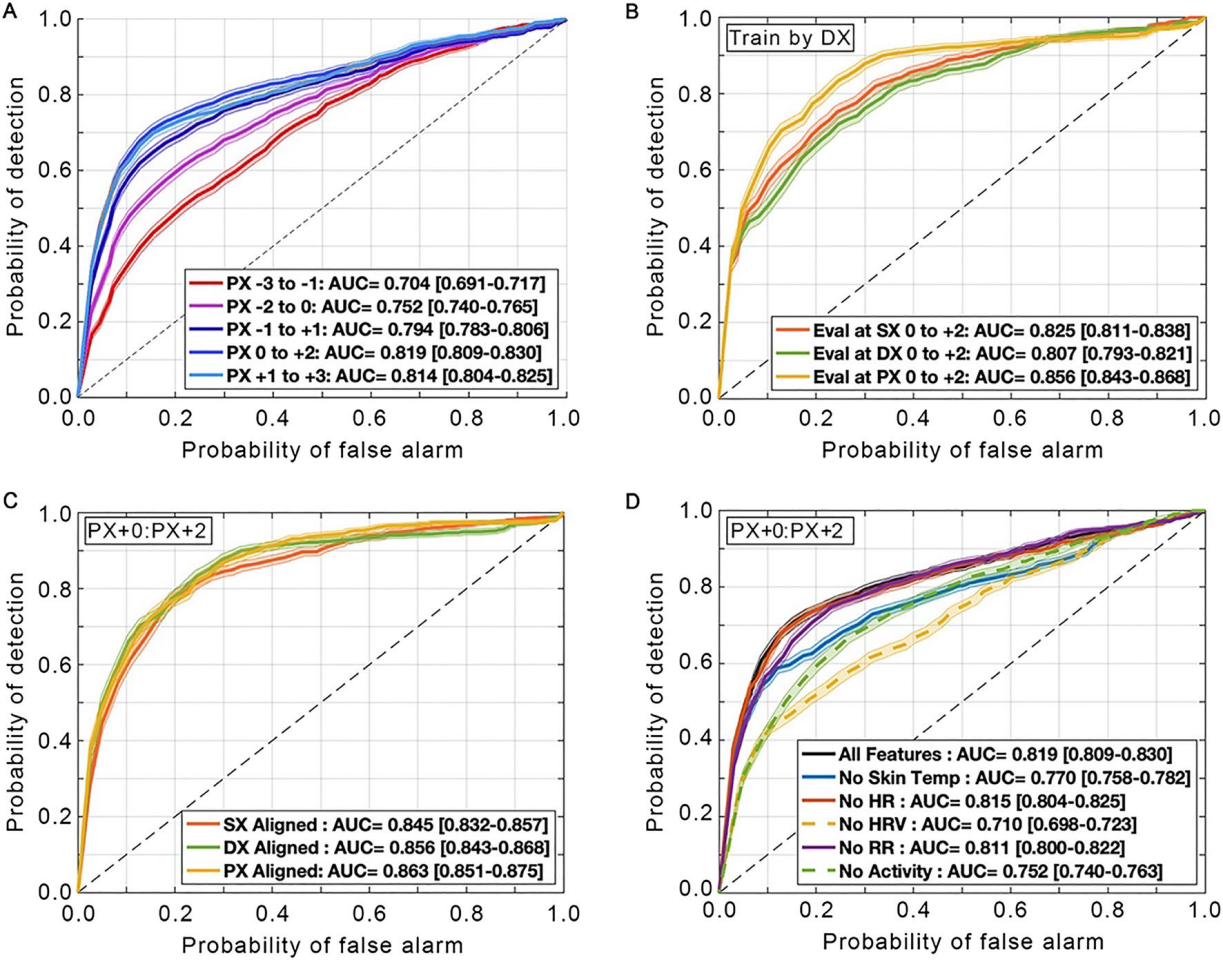
Algorithms aligned by PX can be used to classify COVID-19 infection. Each panel shows a set of receiver operator curves (ROC) with shading indicating  ±  95% CI. PX = date of maximal change from average over the 21-day DX region; SX = date of onset of one of four core symptoms of COVID-19; DX = date of diagnostic testing for COVID-19; HR = heart rate, HRV = heart rate variability, and RR = respiratory rate. Numbers in relationship to PX, SX, and DX refer to number of days before (negative numbers) or after (positive numbers) each of these dates. Models trained by alignment to PX were more accurate as the evaluation window approached PX (**A**; from red pre-PX to blue post-PX; *n* = 73; in all cases, the number of negative training samples was 179,010; the number of positive training samples were: 8678, 9059, 9527, 9719, and 9705, respectively), with a peak accuracy at the window of PX + 0: PX + 2 days. ROC curves generated from models trained by alignment to DX performed best when evaluated relative to PX (**B**; *n* = 41, restricted to the subset of individuals with reliable symptom onset reports). Models trained by alignment to PX, SX, and DX performed comparably when evaluated at PX + 0: PX + 2 days (**C**; *n* = 41). Exclusion of any physiological measure lowers performance, with the ROC AUC dropping the most when HRV was omitted (**D;** *n* = 73).

We identified three key dates that would allow algorithmic training and performance: traditional diagnosis date (the date on which diagnostic testing was performed, DX), initial symptom reporting date (onset of one of four core symptoms of fever, fatigue, dry cough, or unexpected loss of smell or taste, SX), and a novel date of physiological alterations (based on the maximal change from average over a 21-day period in heart rate and respiratory rate, PX). We evaluated models using three-day windows that we defined in relationship to key dates (e.g., DX date through DX date + 2 days). We used machine learning methods to train a classifier algorithm using physiological data to distinguish participants during a period around their diagnosis of COVID-19 versus during a comparison period from the same participants about six weeks prior to developing COVID-19. We successfully trained classifier models on three-day windows relative to PX. We found that ROC AUC increased when we evaluated performance in windows approaching PX (Fig. 2A). Model performance reached peak accuracy 2.75 to 0.75 days ahead of DX (ROC AUC = 0.819 at PX + 0: PX + 2 days, 95% CI [0.809, 0.830]).

To test the null hypothesis that DX provided the best alignment, we re-trained models by alignment to DX. On these models, performance evaluated relative to PX was higher than when evaluated relative to DX or SX (Fig. 2B). Because evaluation aligned to PX yielded the highest ROC AUC, we re-evaluated performance on PX using models trained by alignment to PX, SX, and DX (Fig. 2C), and found that the models performed comparably. Therefore, as PX occurred on average earlier than SX or DX, and as alignment to PX provided higher ROC AUC without an associated cost when training by this alignment, we used models trained by alignment to PX for the remaining analyses.

Importantly, model performance was best when using all physiological data streams. Removing any one data stream reduced performance (Fig. 2D). Including dermal temperature improved ROC AUC from 0.770 to 0.819 (4.9% absolute improvement). Analysis with this complete algorithm provided a sensitivity of 82% and a specificity of 63% (*n* = 73) across the 21-day DX region.

**Figure 3:**
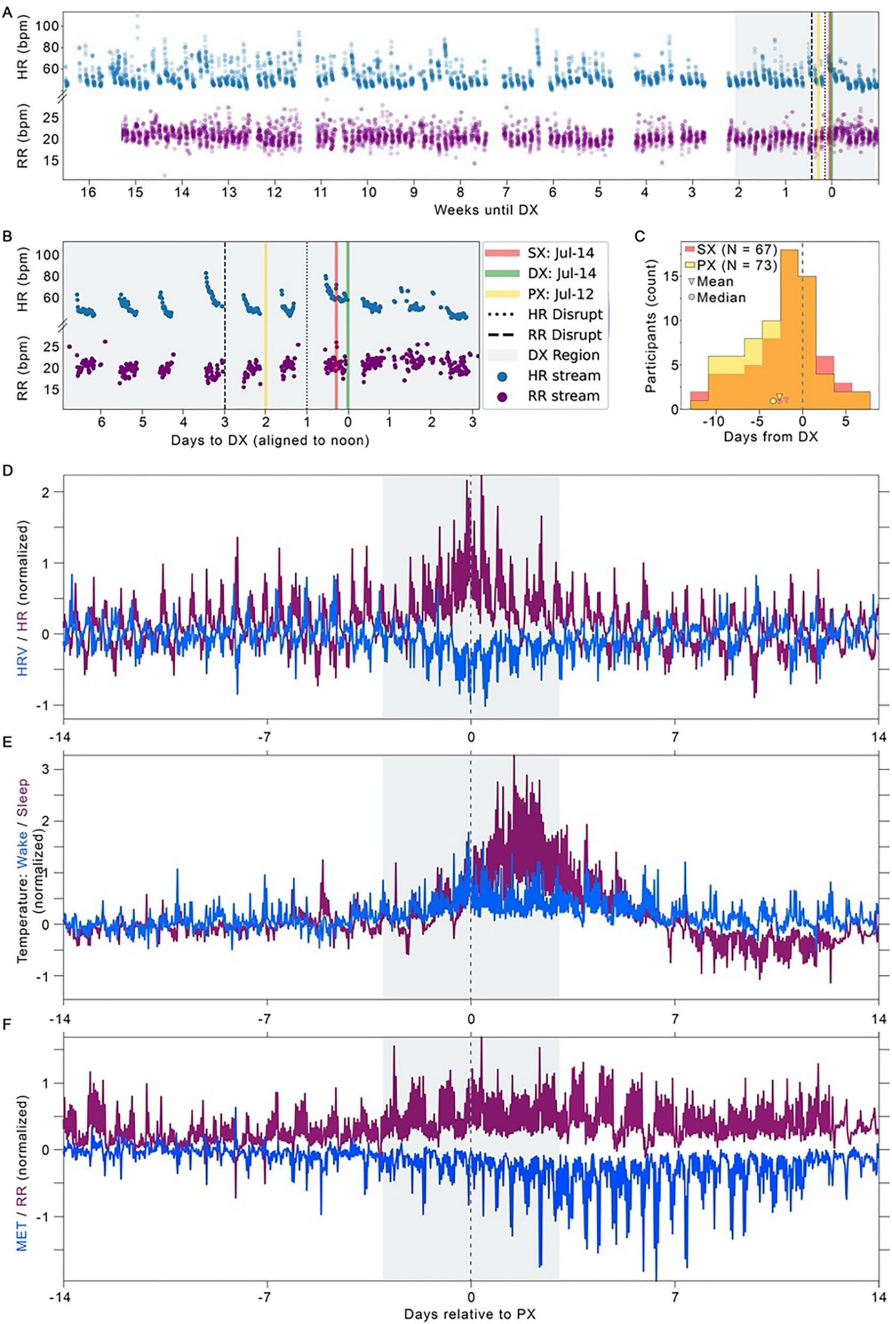
Continuous physiology data allow more precise alignment for machine learning training, sickness profiling. We analyzed continuous heart rate (HR, beats per min, blue) and respiratory rate (RR, breaths per min, purple) within the presumptive illness window of DX-2 weeks: DX + 1 week (grey shaded region) to detect statistical deviations (**A,** dashed lines; zoom in **B**); the average location of the two detections defined PX (yellow). On average, distance from PX to DX was 1 day longer than SX to DX (**C**); SX (*n* = 67) relies on report, and so is missing in some cases when PX (*n* = 73) is present. Profiles of physiological data aligned by PX from the *n* = 73 cohort for heart rate (HR) and heart rate variability (HRV; **D**), dermal temperature during wake and sleep (**E**) and estimated metabolic equivalents (MET) of physical activity and respiratory rate (RR; F). See Fig. 2 and Methods for definitions of DX, PX, and SX.

As shown in Fig. 2B,C, our novel approach for aligning data to PX (Fig. 3A,B) enhanced model performance. We found that SX occurred a mean of 1.98 days earlier than DX, whereas PX occurred a mean of 2.75 days earlier than DX (Fig. 3C). PX occurred on or before SX and DX in most cases (65% and 80%, respectively). Additionally, training aligned to PX improved the AUC by 4.2% compared with training aligned to SX, and by 8.7% when compared with training aligned to DX.

### Visual assessment across data streams

To assess the physiological features that might underly our algorithm’s accuracy, we visualized the mean physiological profile at 30 min temporal resolution across the DX region for all modalities (HR, HRV, RR, MET, and dermal temperature). Alignment by PX revealed increases in dermal temperature, HR, and RR, and decreases in HRV and MET (Fig. 3D–F) around PX, with different data streams revealing different time-courses to apparent recovery (return to baseline ranges). The increase in dermal temperature was more readily apparent during periods of sleep than wake, consistent with our previous work^7^. This pattern of physiological changes highlights the value of alignment by physiological features, which maximizes information gained from including temperature, as well as the potential for added sensor modalities to improve precision disease profiling and recovery monitoring.

### Antibody confirmation of infection status

**Figure 4:**
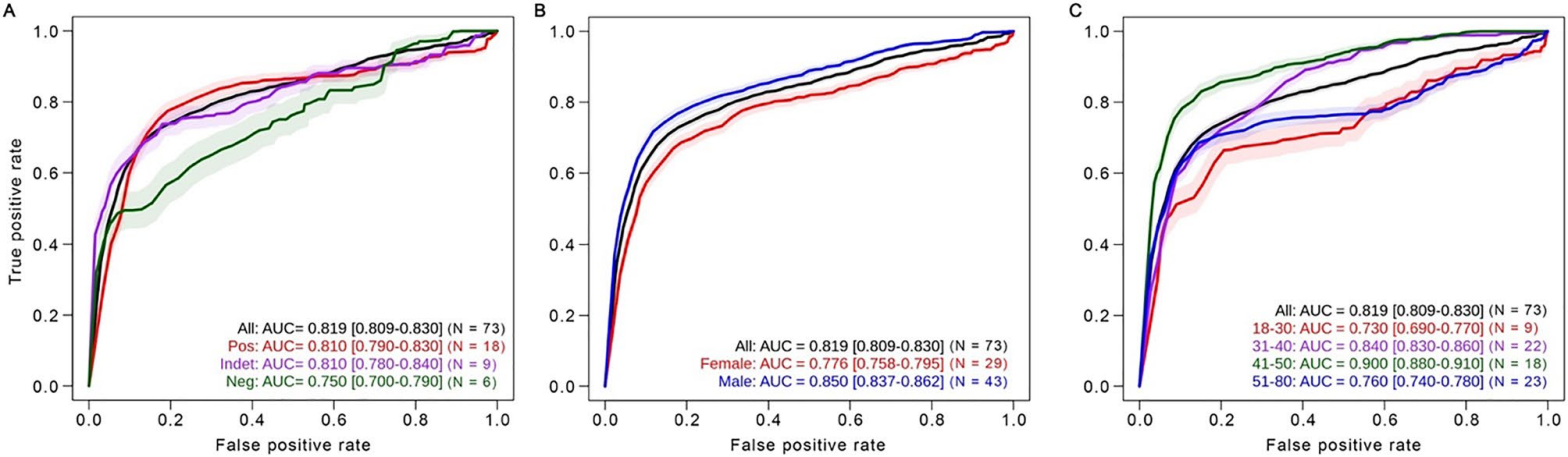
Accuracy changes across different populations. The model trained at PX + 0: PX + 2 showed different performance accuracy (ROC AUC) when we segregated participants by antibody test result (**A**), sex (**B**) and age group (**C**); [95% CI] (N). Each panel uses the participants (*n* = 73) who reported positive diagnostic tests for SARS CoV-2 and were included in algorithm training. Pos = positive, Indet = indeterminate, Neg = negative antibody test. The algorithm performed as expected on individuals with positive antibody tests (red), who were very similar to individuals with indeterminate antibody tests (purple). The algorithm was less accurate for individuals with negative antibody tests (green), consistent with the algorithm showing COVID-19 specificity. The ROC AUC for women was lower than the ROC AUC for men. Age groups showed different levels of overall accuracy that were not merely proportional to N.

During the TemPredict study, we obtained funding for SARS CoV-2 antibody testing using dried blood spots ^9^. We sent antibody testing kits to 10,021 willing participants who met data quality thresholds. Of the 73 participants selected for algorithm training, 33 subsequently had antibody results that became available after algorithm development had been locked. Of these 33 participants, 18 had positive antibody results, 9 had indeterminate results (likely consistent with SARS CoV-2 infection, but less conclusive; Fig. 4A), and 6 had negative results. Some of these may be false negative results as antibody testing on dried blood spots is less sensitive than tests using plasma. However, these antibody testing results may also indicate that some of the self-reported COVID-19 cases in our algorithm training set had false positive COVID-19 diagnostic tests or inaccurately self-reported test results to TemPredict.

To confirm how the algorithm performed in people who we were highly confident had COVID-19, we went back to the 306 participants with reliable self-reported diagnoses of COVID-19, from which we had selected the 73 participants for algorithm training. We identified an additional 10 participants (separate from the 73 included in algorithm training) with a positive dried blood spot antibody test and adequate physiological data around the time of their DX to apply our algorithm. In this new set of participants, we ran detections in the DX + 0 to DX + 2 range to ensure comparability to our peak accuracy in the 73 previous individuals (Fig. 2A), and found the algorithm had an overall ROC AUC of 0.819 (95% CI [0.809, 0.830]), with a specificity of 80% and sensitivity of 90% (*n* = 10) across the 21-day DX region.

### Effects of heterogeneity

A key promise of wearable technology is the ability to tailor algorithms for different populations. We therefore assessed consistency of our algorithm’s performance across sex assigned at birth and age. We found that the algorithm trained across all participants had a 6.7% lower ROC AUC in women than in men (Fig. 4B). Similarly, we observed differences in algorithm performance by age, with the biggest difference in accuracy occurring between people in their 40 s (ROC AUC = 0.900, 95% CI [0.88, 0.91], *n* = 18) and people younger than 30 (ROC AUC = 0.730, 95% CI [0.69, 0.77], *n* = 9; Fig. 4C). These findings suggest that although wearable technology may provide important information with which to develop illness detection models, algorithm development will require training using diverse populations before it can be reliably deployed over diverse populations.

## Discussion

We developed an algorithm to identify COVID-19 onset using data collected by a commercially available wearable device. The resultant algorithm had high sensitivity (82%), with moderate specificity (63%). In developing this algorithm, we placed greater emphasis on sensitivity than on specificity, as our goal was to develop an algorithm that could effectively identify individuals who should obtain laboratory-based diagnostic testing. In this context, lower sensitivity would result in fewer people with potential COVID-19 receiving diagnostic testing, which poses a more serious problem in most screening settings than lower specificity, which would result in more people without COVID-19 receiving diagnostic testing. These findings suggest that algorithms derived from physiological data collected by wearable devices could help individuals make decisions about seeking diagnostic testing. This may be particularly important in the context of a disease like COVID-19, in which many individuals cannot easily identify whether they are infected based on symptoms or a single temperature assessment^10^. For example, the sensitivity of thermal screening to detect individuals with COVID-19 has been reported to be only about 10%^11^.

Our findings make several important contributions to a growing body of literature^4–6,12,13^ documenting the utility of wearable devices in illness detection. Importantly, we believe this is among the first efforts to report on an algorithm based on physiological data collected by a wearable device that has included objective validation of disease status using laboratory antibody testing rather than solely relying on self-report. Although antibody testing using dried blood spots, as done in this study, has limitations due to reduced test sensitivity, we performed rigorous assessment of algorithm performance in a separate validation sample of individuals with both positive antibody tests and self-reported COVID-19 diagnoses. This analysis provided important support for the performance of the algorithm, which achieved an even higher ROC AUC in the antibody-confirmed validation sample than we observed in the training sample. This provides important confirmation that the initial algorithm assessment was unlikely to overestimate algorithm performance due to overfitting data or including individuals who did not actually have COVID-19.

Our physiological measures included continuous temperature data, which are not widely available on wearable devices, and have not been used in other efforts that have used physiological data from wearables to identify individuals who had COVID-19^4–6,12,13^. Importantly, we found that temperature played a substantial role in improving algorithm performance. We also introduced a novel physiologically derived onset label characterized by physiological alteration (PX). PX allowed us to capitalize on the continuous nature of wearable device data to improve upon alignment from the more traditional symptom date (SX) and diagnosis date (DX) labels. Alignment of multimodal physiological time series data by PX allowed us to generate high-resolution profiles of physiological change across COVID-19 illness and into recovery. Together, these approaches highlight novel methods by which wearable devices can support future precision medicine and public health interventions.

A few important limitations of our data deserve mention. First, although we carefully assessed the self-reported history of COVID-19 and only selected participants who described obtaining positive PCR or antigen test results for the training dataset, we may have included some individuals who either misunderstood or misreported their COVID-19 diagnostic test results, or who had false positive diagnostic tests. Six of the 33 individuals in the training dataset who completed study-provided COVID-19 antibody testing using dried blood spots had negative antibody tests (these results became available after algorithm development), however, the reduced sensitivity of dried blood spot tests means some of these likely represent false negative antibody tests. An initial concern with including individuals who did not actually have COVID-19 in the training dataset is that this would provide a misleadingly high estimate of the algorithm’s performance in individuals with COVID-19. We mitigated this concern by completing analyses in a separate validation sample with positive antibody tests and these analyses demonstrated slightly better algorithm performance. Second, although our data suggest that algorithm performance might be optimized by modifying it based on factors like sex and age, we did not have an adequate sample size to do this well in smaller subsets of participants. This area deserves additional attention in future efforts. There are meaningful racial disparities at the intersection of wearables and health that must be addressed by population-wide data collection and deployment of solutions emerging from this space^14^. Third, we developed this algorithm using retrospective approaches, and testing feasibility and utility of real-time deployment is a key next step. Fourth, our data did not include individuals who had been vaccinated against COVID-19, and we assume that given the time period of our study (March 2020 through November 2020), our positive cases were infected with the original D614G variant of SARS CoV-2. Whether this type of algorithm performs differently in vaccinated individuals or individuals infected with different SARS CoV-2 variants requires further assessment. Finally, given the complexity and the coupled nature of many physiological systems^15–19^, we expected that different physiological modalities would add new information, and confirmed this by including continuous temperature data. There may be limits to diagnostic specificity derived from physiological time series (e.g., discerning influenza cases from COVID-19), but this remains to be tested.

Future work should focus on algorithm development in diverse populations so as to optimize accuracy in heterogenous populations. Such work should use study designs that allow for identification of physiological signatures in both baseline periods of health and periods of illness. Future analytic approaches should further compare the utility of within- versus between-individual comparison for model training; novel methods may find that integrating both approaches yield better tools. Systems that integrate passive collection of physiological information in tandem with self-report information, like that which we developed here, could support rapid deployment of early detection methods in the future that individuals can access independently. Although algorithms derived from wearables have the potential to address future epidemic responses^8^, clarifying regulatory models and privacy protections for these emerging tools is critical^20^. Without these clarifications, scientific capacity may outpace the scaffolding needed to ensure equitable, rapid, and successful deployment.

## Methods

### Study design

We began recruiting participants on March 19, 2020. Recruitment was rolling and stopped on September 23, 2020. We first consented participants to participate through August 30th, 2020. Due to the continuation of the pandemic, we contacted participants with the invitation to consent to extend their study participation to November 30th, 2020.

We recruited adults from the broader population who already possessed Oura Rings by sending them invitations within the Oura App on their smartphones. Prospective participants could tap on this invitation, which linked to the UCSF consent survey online. We did not have a recruitment ceiling for participants who met eligibility criteria and possessed their own Oura Rings. We recruited frontline healthcare workers at participating sites by enlisting leadership at each institution and obtaining IRB review at each institution. We mailed sites recruitment materials, including study flyers and Oura Ring sizing kits, which contained plastic rings for healthcare workers to try on to determine their size. These kits also included instructions about how to reach the UCSF consent survey online. In this survey, prospective participants could review and download the study consent form, indicate their Oura Ring size, and enroll in the study. All participants provided informed consent to participate in the study. Our target recruitment for healthcare workers to whom the study would provide Oura Rings was *n* = 3,400. We recruited a subset of participants (*n* = 10,021) located within the U.S. to complete mail-based antibody testing using dried blood spot (DBS) cards.

### Sites

We recruited participants at the following healthcare sites: The University of California San Francisco hospitals at Mission Bay and Parnassus; Zuckerberg San Francisco Hospital and Trauma Center; Stanford Medical Center; Santa Clara Valley Medical Center, Northwestern McGaw Medical Center; Beth Israel Deaconess Medical Center-Harvard Medical School; Stony Brook University Renaissance School of Medicine; Stonybrook Medical Center; Weill Cornell Medicine; New York-Presbyterian Queens; New York-Presbyterian Brooklyn Methodist Hospital; University of Miami Health System; University of Texas Southwestern Medical Center Dallas; Tufts Medical Center; Jamaica Hospital Medical Center; University of California Los Angeles Medical Center; Boston Medical Center; Kaiser Permanente San Diego Medical Center; Florida Atlantic University, and American Medical Response (ambulance). Participants who already owned an Oura Ring and who we recruited from the existing user base via the Oura app were distributed globally.

### Antibody testing

We aimed to recruit 10,000 participants to participate in DBS COVID-19 antibody testing using the following selection criteria, in the following order of priority: (a) Reported (via daily survey) a positive COVID diagnosis; (b) had high illness probability based on symptoms reported in daily survey and had extensive Oura Ring data available (minimum 100,000 observations and maximum 2 day gap); (c) located in COVID-19 hotspot ZIP code (40-mile radius of ZIP 10010 or 48206) at the time of consenting; (d) located in top 50 hotspot counties; (e) with Oura Ring data going back to at least April 15; (f) moderate illness probability based on reported daily symptoms and minimum of 100,000 Oura Ring data observations available; (g) modest illness probability based on reported daily symptoms and Oura Ring data spanning the period of March to May; (h) remaining participants, sorted by illness probability.

### Participants

Eligible participants were frontline healthcare workers at one of several participating healthcare institutions whom the study provided with Oura Rings, or adults who possessed an Oura Ring of their own that they used for participation. Eligible participants were at least 18 years of age, possessed a smartphone that could pair with their Oura Ring, and could communicate in English. The University of California San Francisco (UCSF) Institutional Review Board (IRB, IRB# 20-30408) and the U.S. Department of Defense (DOD) Human Research Protections Office (HRPO, HRPO# E01877.1a) approved of all study activities, and all research was performed in accordance with relevant guidelines and regulations and the Declaration of Helsinki. All participants provided informed consent (electronic). We did not compensate participants for participation.

### Procedures

Prospective participants visited a survey hosted on the UCSF Qualtrics platform, and after reviewing information about the study, could download a PDF of the study consent form, and if they wished to enroll, provided digital consent. Participants then completed a baseline survey, wherein they entered demographic and health information (see “Measures”). We asked participants who were waiting for an Oura Ring to download the Oura App, and upon receiving an Oura Ring in the mail, to pair the Oura Ring with the Oura App and opt to share their Oura data with UCSF (from within the Oura App). We asked participants who already possessed an Oura Ring to share their Oura data with the research team from within the Oura App. Participants were presented with a daily in-app message that linked to a brief survey that asked them to report whether they were experiencing potential COVID-19 symptoms and whether they had received COVID-19 testing or diagnoses (see “Measures”). We also asked participants to complete monthly surveys that included questions about health behavior and diagnoses, mental health and psychological stress, and COVID-19 exposure. In June and July of 2020, we mailed the first of two DBS cards to participants who consented to complete mail-based antibody testing. We mailed participants a second DBS card roughly 8 weeks after we received their first completed DBS card by mail (see “Measures”).

### Measures

We collected the following measures.

#### Baseline self-report survey

Participants reported on demographic factors including age, biological sex, race/ethnicity, educational background, anthropometric information, country and state of residence, and other factors that are not the focus of this manuscript.

#### Daily self-report surveys

Participants reported on whether they had experienced any of the following symptoms since they last completed the survey: fever, chills, fatigue, general aches and pains, dry cough, sore throat, cough with mucus, cough with blood, shortness of breath, runny/stuffy nose, swollen/red eyes, headache, unexpected loss of smell or taste, loss of appetite, nausea/vomiting, and/or diarrhea. Participants also reported on whether they had received any new testing results for COVID-19 and could indicate the type of testing (nasal or oral swab specimen, antibody blood test, saliva/spit specimen, stool specimen, or other with the ability to specify) and the date they provided the test specimen. Participants also reported on the results of their test (positive, negative, or indeterminate).

#### Monthly self-report surveys

Participants reported on any medical diagnoses (including COVID-19), as well as COVID-19 exposure, health behavior, alcohol and drug use, prescription medication information, and mental health. For each diagnosis they endorsed, they also reported on whether their diagnosis was confirmed with testing. If a COVID-19 or flu diagnosis was confirmed with testing, participants answered questions that were identical to those in the daily survey (type, date, and results of testing).

#### Dried blood spot (DBS) antibody testing

To obtain specimens for SARS CoV-2 antibody testing, we mailed kits for obtaining dried blood spots to 10,021 participants (TropBio Filter Paper Disks, Cellabs). We prioritized sending kits to willing participants who had higher quality Oura Ring data, completed more symptom surveys, who were located in the U.S. (due to cost and regulatory complexity of international shipping), and who were within specific geographic locations (greater prevalence of and/or exposure to SARS CoV-2). The collection kit included tabs for obtaining up to six dried blood spots. We instructed participants to dry their blood spots overnight before returning by mail in plastic specimen bags containing a desiccant. We processed DBS with eluent and tested using the Ortho Clinical Diagnostics VITROS® SARS CoV-2 Total Assay^9^. To validate testing using dried blood spots we performed several steps, including preparing dried blood spots from whole blood obtained from individuals diagnosed with COVID-19 who tested positive on serum testing for comparison to testing methods on which the assay was originally developed and validated. In validation testing using these dried blood spots, we found the use of dried blood spot sample collection reduced the sensitivity of the antibody testing to about 90% compared with standard sample collection methods that performed the same assay on plasma. For comparison, the sensitivity of the Ortho VITROS ® SARS CoV-2 Total Assay is reported to be 98.8% with patients confirmed to be SARS-CoV-2 positive by PCR^21^ using serum specimens. While the assay normalized signal-to-cutoff (S/CO) ratios provide clear separation between reactive and non-reactive specimens on serum specimens, we found some overlap between S/CO results on DBS specimens from individuals with COVID-19 based on PCR testing or serum SARS CoV-2 antibody testing with the Ortho VITROS ® SARS CoV-2 Total Assay. We therefore designated an indeterminate test range where there was evidence of overlap in S/CO values between individuals with and without prior SARS CoV-2 infection.

#### Oura ring data

All participants wore the Oura Ring Gen2 (ouraring.com), a commercially available wearable sensor device (Oura Health, Oulu, Finland), on a finger of their choosing. The Oura Ring connects to the Oura App (available from the Google Play Store and the Apple App Store) via Bluetooth. Users wear the ring continuously during daily activities in both wet and dry environments. The Oura Ring assesses temperature by using a negative temperature coefficient (NTC) thermistor (resolution of 0.07 °C) on the internal surface of the ring. The sensor registers dermal temperature readings from the palm side of the finger base every 60 s. The Oura Ring assesses heart rate (HR), heart rate variability (HRV), and respiratory rate (RR) by extracting features from a photoplethysmogram (PPG) signal sampled at 250 Hz. The Oura Ring calculates HR and HRV in the form of the root mean square of the successive differences in heartbeat intervals (RMSSD) at 5 min resolution. The Oura Ring also estimates RR at 30 s resolution. The PPG-derived metrics (HR, HRV, RR) are calculated from inter-beat intervals (IBI), which are only available during periods of sleep. Tri-axial accelerometers estimate activity metrics as metabolic equivalents (MET) reported at 60 s resolution during both sleep and wake periods and sleep stages at 5 min resolution. The Oura Ring generates all of these metrics, which we will refer to as the five data streams, on device. The Oura Ring does not continuously record or store PPG for analysis.

### Variable creation

We created several variables for these analyses as follows.

#### Diagnosis determination (DX)

We identified COVID-19 cases based on data from daily and monthly surveys, with confirmation from study-provided SARS-CoV2 antibody testing on dried blood spot (DBS) specimens when available (*n* = 3664 participants had SARS-CoV2 antibody results from submitted specimens at the time of case definition). A total of *n* = 704 participants self-reported having COVID-19.

#### Confirmed positive cases

These were participants who reported a positive COVID-19 test result on an oral or nasopharyngeal swab, saliva, stool, or antigen test*.* We identified the diagnosis date as the earliest reported positive test date across surveys to capture the first test positive date. Confirmed positive cases did not provide discordant reports across survey reports or test types.

#### Confirmed negative cases

These were participants who tested negative on study-provided DBS antibody testing and who did not report positive COVID-19 test results in any study survey.

#### Test ambiguous cases

These were participants who had a negative result on study-provided DBS antibody testing (*n* = 9) or who self-reported a negative antibody test result after a reported positive swab, saliva, antigen, or stool test (with an 11-day buffer to allow time for seroconversion). These were suspected false positive tests.

#### Survey ambiguous cases

These were participants who reported conflicting results for the same test in the same 4-day period across different survey types (which we considered to be reporting errors that we reconciled by contacting participants to confirm reporting).

#### DX-generated case lists

At the time we generated the positive case lists, *n* = 210 participants reported a positive swab test on a daily survey, and an additional 108 reported a positive swab test on monthly surveys (that they did not report in daily surveys); *n* = 4 reported positive saliva tests (without a prior swab test). Where reports were conflicting (where test results differed for the same test type reported within an interval ± 4 days across different survey instruments), or for participants who reported a positive COVID-19 test in the very early study weeks before test type questions were added to the daily survey, we followed up with participants to obtain additional testing-related information and completed this follow up with *n* = 113 participants. During follow-up, two participants reported a positive swab test and one reported a positive antigen test that they had not previously reported on another survey type. After removing test ambiguous (*n* = 11) and survey ambiguous (*n* = 7) cases, the final list of COVID-19 confirmed positive cases included 306 participants positive by swab (*n* = 302), saliva (*n* = 3), or antigen (*n* = 1) test. Among these, we confirmed *n* = 45 with positive study-provided DBS antibody testing.

#### DX region

We defined a time of probable COVID-19 infection (*DX region*) by proximity to the confirmatory COVID-19 test date as 14 days before (DX – 14), and 7 days after (DX + 7) the testing date.

#### Self-report (per daily surveys) symptom onset date determination (SX)

Daily surveys include a list of symptoms that may be endorsed each day. To determine the date of symptom onset (SX), we focused on four core symptoms associated with COVID-19: fever, fatigue, dry cough, and unexpected loss of smell or taste. For a window surrounding the diagnosis date, spanning 14 days prior to DX through 7 days post-DX, we looked for participants who self-reported a transition from “no symptoms” to one or more core symptoms with no more than 2 consecutive missed survey responses in the vicinity of this transition. We defined symptom onset as the first day in the DX region in which participant reported a core symptom *following one or more days* of “no symptoms.” We considered participants who completed the daily surveys during the window around the diagnosis date, but who did not endorse any of the four core symptoms, to be asymptomatic. We did not attempt to establish symptom onset dates for participants who completed fewer than three symptom surveys in the DX region, or those who did not endorse the “no symptoms” option at least once prior to the first reported symptom.

#### Physiological (per Oura data) disruption date determination (PX)

To develop a method for imputing illness onset for individuals with incomplete or missing symptom reports—and, more generally, to decide *where* in the time series to search for informative, infection-related patterns—we designated a data-driven, physiological disruption (PX) date for each participant of interest (*n* = 73). We derived PX from two of the five Oura data streams (HR and RR time series). We compared the resultant PX values with 1) SX for those 41 individuals with the highest-confidence symptom self-reports and 2) the dates of coincident, physiological changes across the other three streams for all these 73 participants.

To impute a single date for physiological disruption, we first designated 21-day, individually curated baseline periods for a subset of participants who tested positive for COVID-19 (*n* = 73; see COVID-19 infection data availability; see also Algorithmic description). We then computed means over all the values taken by the HR and RR time series in these baseline periods. We compared the baseline means to each of the 21 mean *daily* heart rate and mean *daily* respiratory rate values that characterized the respective individuals’ DX regions; this allowed us to assign dates for the *maximal observed heart rate and respiratory rate deviations* in the extended neighborhood surrounding illness onset. Maximal deviations in one’s daily heart rates need not occur simultaneously with those of their respiratory rates. Thus, in keeping with intentions to designate a single physiological (per Oura data) disruption date, per individual, we assigned as PX the temporal midpoint between the maximal HR- and maximal RR-derived deviations.

We observed that substantial deviations in both the average daily heart rates and respiratory rates in the vicinity of the diagnosis date (DX) were overwhelmingly of finite duration—typically lasting for several days—among the aforementioned subset of participants (see COVID-19 infection data availability). We also observed shorter-duration (i.e., overnight, etc.) fluctuations in the HR and RR series in the days surrounding DX, but these fluctuations did not necessarily reflect infection-related disruptions. In order to penalize the influence of the latter on PX determination, and to instead emphasize the most salient, trend-like deviations (e.g., a clear rise in the respiratory rate over three consecutive days), we operationalized our PX imputations into several steps.

First, we treated each 21-day sequence of absolute deviations (for heart rate and respiratory rate) as its own time series signal. We then identified the *peaks* associated with each such signal, according to criteria for numerical analysis defined by the *SciPy 1.6.3* package for Python. We did not impose constraints regarding the minimal threshold for peak detection, relative peak heights, or distances by which successive peaks should be separated; we did exclude “peaks” corresponding to the first two days (DX—14 to DX—12) of the 21-day sequence, as it would take at least three observations to establish coherent trends. The highest peaks represented the maximal deviation dates for an individual’s heart rate and respiratory rate signals. In cases where *SciPy* did not detect peaks, or detected peaks only within the first two days of a given signal, we assessed maximal deviation dates from the full subset of 21 values for that signal.

### Oura data preparation

The Oura Ring records five physiological metrics (data streams) on the scale of minutes. For the present analyses, we aggregated data from each of the five streams within 30-min, consecutive time intervals that overlapped by 15 min. We chose these time frames to balance computational resolution (i.e., the inherent tradeoff between an ability to work with fine-scale features and data-architecture costs and considerations) with expectations based on physical and physiological limitations for the observability of illness-related changes. Within each time interval, we extracted a set of summary statistics from the available physiological metrics, including their mean, standard deviation, and 25th and 75th percentile values. For example, the Oura dermal temperature metric, *T*, is natively sampled once per minute; we aggregated the (up to 30) temperature samples in each 30-min interval to compute the temperature-derived variables *T*_*mean*_, *T*_*std*_, *T*_*per25*_, and *T*_*per75*_ (temperature mean, standard deviation, 25th and 75th percentiles, respectively). These “derived” variables compactly summarized all dermal temperature measurements falling within the respective intervals, replacing the high-resolution temperature time series in our algorithmic computations.

Potential artifacts in wearable data include records saved during non-active wear times (e.g., elevated temperature readings, saved while an Oura Ring is charging). We preprocessed the dermal temperature and MET data to determine times when participants’ rings were actively worn, versus non-wear times, by comparing MET values against a fixed threshold of 0.5. We treated values below this threshold as non-wear and discarded both MET and dermal temperature measurements during these non-wear periods.

### COVID-19 infection data availability

Beginning with a cohort of 306 participants for whom there was a confirmed self-reported diagnosis of COVID-19 based on a positive oral or nasal swab, saliva, or stool specimen tested using PCR or antigen assays (see “Diagnosis determination (DX)”), we identified participants for inclusion in the training dataset. We selected individuals for whom we had Oura data available on at least 20 days (consecutive or not) that would be usable as baseline (at least 17 days prior to DX) and had at least 7 days of data prior to DX and 14 days following DX. Additionally, we excluded individuals who reported 4 or more concurrent symptoms (see “Self-report (per daily surveys) symptom onset date determination (SX)”) or exhibited dermal temperatures above 38 degrees Celsius in the baseline period, so as to screen potential confounding illness. For algorithm training, we restricted analysis to 73 participants that met the above criteria and had complete heart rate, respiratory rate, and temperature data. For independent validation, we considered only participants outside this training set with confirmatory antibody results following their DX and physiology data available for i) at least 13 of their baseline days, and ii) no less than 20 of their DX region days. Participants in the independent validation set therefore met minimum data requirements similar to those described above, but we omitted restrictions on symptoms and elevated dermal temperatures in the baseline period. Ten participants met the secondary criteria for inclusion in the independent validation set.

### Algorithmic description

We created a machine learning pipeline that detected physiological features distinguishing COVID-19 illness from non-illness. This pipeline had 3 constituent parts: 1) data processing module, 2) short-time classification and detection module, and 3) post-detection "trigger” logic module.

The data processing steps were to a) gather and b) normalize individuals’ data. We refer to the compressed time series described in “Oura data preparation” as data *sketches.* The data sketches consisted of aggregated statistics extracted from successive 30-min intervals; we created data sketches for each of the five physiological data streams. Additionally, we generated several new variables that capture longer-duration *trends* by applying moving-average filters across the data sketches. This allowed us to learn illness-related features that occur over multiple time scales. Measurements of physiological signals may have distinct characteristics during wake versus sleep; therefore, for trend assessment, we calculated separate variables from measurements taken during wake and sleep. We set our moving-average filters to calculate 1-, 2- or 3-day trends from the “asleep” and “awake” data sketches. For dermal temperature, variables included a 3-day moving average and moving standard deviation of *T*_*per25*_ during wake intervals, and a 3-day moving average of *T*_*per75*_ during sleep intervals. For activity, our primary trend variable was a 3-day moving average of the 75th-percentile MET value during wake intervals. The full set of variables supplied as inputs to the pipeline are listed in Supplementary Table 1.

Baseline physiology can vary greatly between individuals. We therefore normalized all physiological variables (data sketches and trend variables) according to each individual's baseline values. To do so, we subtracted individuals’ baseline means and divided by their baseline standard deviations (z-score). We nominally estimated baseline mean and standard deviation values using the 21-day baseline period data (see Physiological (per Oura data) disruption date determination (PX)). In several cases, missing data precluded the use of the full 21 days for baseline estimation, and we therefore designated an alternative baseline period for normalization for these cases. Specifically, in these cases, we estimated the baseline mean and standard deviation using available baseline data in the 49 days prior to DX.

We trained a set of five Random Forest models on the normalized data sketches and trend time series. The classifier training samples consist of data from overlapped 30-min intervals from individuals assigned to the training set and each of the five models were differentiated by considering distinct time frames as the positive class. The set of trained classifier models were then used to predict a preliminary score at each interval assessing whether the individual appeared sick, and a *detection score* was computed as the fraction of sick intervals out of the last 48 intervals (i.e., nominally 12 h of real-world data), for each interval.

The ground-truth target labels for this classifier were provided by treating all time intervals in each individual as not sick (“negative” training samples) across up to 73 days pre-COVID (from −90 days to −17 days with respect to PX) and sick on five distinct time frames in the vicinity of PX as progressive phases of illness (“positive” training samples).

The five Random Forest models were trained such that each model encompassed a different positive time frame near PX. The negative training samples were held constant for all five models. The first of these models was trained on data sketch and trend variable values drawn from the range PX − 3 to PX − 1; the second covered PX − 2 to PX; the third, PX − 1 to PX + 1; fourth, PX to PX + 2, and fifth PX + 1 to PX + 3. In this way, we learned patterns relevant to infection *at each of several “early-stage” time frames* in the vicinity of illness onset. For example, one of the earliest signs of oncoming illness in our data may be encoded in aberrant nightly heart rates while temperature disruptions arise as a more important sign as the disease progresses through the incubation period. Rather than build in constraints based on clinically recognized illness patterns or prior knowledge from previous research, we allowed the classifier architecture to discern patterns directly from data. Once patterns distinguishing illness from non-illness were identified in each of the five timeframes independently, via training, we ran all 5 classifiers concurrently to search for instances of those patterns during testing, as noted above. The classification and detection process ran continuously across each individual’s time series data. This basic architecture is adapted from previous work on pre-symptomatic detection of infection using animal data sets^22^.

To flag individuals as potentially infected, we carried out several post-detection “trigger” operations on the five sets of detections scores that were reported by the classification algorithms. First, we created a new set of scores, at the same 30-min resolution, by computing their envelope (maximum score across the five classifier models). This served to make salient all those places in the time series where *any* of the learned feature sets would suggest illness. Next, we binned all the detection scores for a given individual, summing all the values at the original 30-min resolution values to form new, aggregate scores that we could associate with each 24-h window. These daily, aggregated scores were compared against a fixed threshold (here, a value of 10) to determine whether our pipeline would pronounce each 24-h bin as a “trigger” opportunity (reputed “sick day”).

### Performance evaluation

The detection performance of our pipeline was evaluated via a five-fold cross validation using data from the identified training cohort (*n* = 73). We calculated ROC curves and their corresponding AUCs using the short-time detection scores generated at 30-min intervals with 15-min overlap. We evaluated the ROC curves against negative and positive ground-truth target labels as defined above (see “Algorithmic description”).

Where we report the ROC curve evaluations for DX rather than PX, we defined the positive labels as DX − 3 to DX − 1, DX − 2 to DX, and so on; and we applied the same method for SX performance evaluation. We report the 95% confidence interval with all AUCs where we assumed that the data points were normally distributed due to the large number of datapoints represented in each curve (see Supplemental Table 2). We assumed the degrees of freedom used in the confidence intervals to be half the total number of datapoints to account for the overlap of the time series data.

Sensitivity and specificity estimates have been reported based on the outcome of the post-detection trigger logic at the fixed threshold. We evaluated these outcomes to assess which individuals would have been triggered, at least once, within their DX regions. The 21 days defined as DX region formed a basis for sensitivity estimates and the 21 days of baseline from 6 to 3 weeks prior to DX formed a basis for specificity estimates.

## Data availability

Oura’s data use policy does not permit us to make the data available to third parties without approval. Therefore, those seeking to reproduce our findings should contact Ashley Mason, PhD, and Benjamin Smarr, PhD for an online application to access the study data portal. This application process will require requesters to make a written commitment expressing agreements to not duplicate data, to not share data with third parties, and/or other confidentiality precautions. Distribution of the classifier code is limited by the Department of Defense and therefore it cannot be shared. However, several novel aspects of the code that were introduced in this body of work are available in the study data portal.

## Supplementary Information

Supplementary Information.
