# Supplementary material for: Detection of COVID-19 using multimodal data from a wearable device: results from the first TemPredict Study
Source: Sci Rep. 2022 Mar 2;12:3463. doi: 10.1038/s41598-022-07314-0 (PMC8891385; doi:10.1038/s41598-022-07314-0)
Supplement: Supplementary file 1 — Supplementary Information. [file 41598_2022_7314_MOESM1_ESM.docx]

Supplementary Table S1.

| **Feature Name** | **Description** |
| --- | --- |
| HR_mean_ | Mean heart rate in a 30-min interval (sleep only) |
| HRV_per75_ | 75^th^ percentile of HRV samples in a 30-min interval (sleep only) |
| IBI_per25_ | 25^th^ percentile of IBI samples in a 30-min interval (sleep only) |
| RR_per75_ | 75^th^ percentile of RR samples in a 30-min interval (sleep only) |
| MET_per75_ | 75^th^ percentile of MET samples in a 30-min interval |
| T_per75_ | 75^th^ percentile of dermal temperature samples in a 30-min interval |
| PercentSleep1Day | Moving average of data samples classified as sleep in a 1-day window (updated every 30 min) |
| TSleepMean3Day | Moving average of T_per75_ during asleep states only, over a 3-day window (updated every 30 min) |
| TWakeMean3Day | Moving average of T_per25_ during awake state only, over a 3-day window (updated every 30 min) |
| TWakeStddev3Day | Moving standard deviation of T_per25_ during awake state over a 3-day window (updated every 30 min) |
| METWakeMean3Day | Moving average of MET_per75_ during awake state over a 3-day window (updated every 30 min) |

Full list and description of the features used by the random forest classifiers.

Supplementary Table S2. Number of data samples used to calculate the ROC curves and their corresponding 95% confidence intervals

| **Figure** | **Category** | **# Subjects** | **# Datapoints –**  **True Positive Rate** | **# Datapoints –**  **False Positive Rate** |
| --- | --- | --- | --- | --- |
| 2A | PX -3 to -1 | 73 | 4877 | 109306 |
| 2A | PX -2 to 0 | 73 | 5226 | 109306 |
| 2A | PX -1 to +1 | 73 | 5528 | 109306 |
| 2A | PX 0 to +2 | 73 | 5750 | 109306 |
| 2A | PX +1 to +3 | 73 | 5739 | 109306 |
| 2B | Train PX/Eval SX | 41 | 5448 | 109558 |
| 2B | Train PX/Eval DX | 41 | 5594 | 106602 |
| 2B | Train PX/Eval PX | 41 | 5750 | 109306 |
| 2C | All Features | 73 | 9719 | 179010 |
| 2C | No Skin Temp | 73 | 9719 | 179010 |
| 2C | No HR | 73 | 9719 | 179010 |
| 2C | No HRV | 73 | 9719 | 179010 |
| 2C | No RR | 73 | 9719 | 179010 |
| 2C | No Activity | 73 | 9719 | 179010 |
| 4A | All | 73 | 9719 | 179010 |
| 4A | Positive | 18 | 2496 | 45258 |
| 4A | Indeterminant | 9 | 1351 | 24008 |
| 4A | Negative | 6 | 718 | 15113 |
| 4B | All | 73 | 9719 | 179010 |
| 4B | Female | 29 | 3873 | 70426 |
| 4B | Male | 43 | 5762 | 106886 |
| 4C | All | 73 | 9719 | 179010 |
| 4C | 18-30 | 9 | 1100 | 19552 |
| 4C | 31-40 | 22 | 2995 | 54855 |
| 4C | 41-50 | 18 | 2418 | 46499 |
| 4C | 51-80 | 23 | 3122 | 56406 |

Details on the number of data samples used to calculate the ROC curves and their corresponding 95% confidence intervals.
